# Impact of Arsenite on the Bacterial Community Structure and Diversity in Soil

**DOI:** 10.1264/jsme2.ME15093

**Published:** 2016-02-20

**Authors:** Dian-Tao Dong, Shigeki Yamamura, Seigo Amachi

**Affiliations:** 1 Graduate School of Horticulture, Chiba University648 Matsudo, Matsudo-city, Chiba 271–8510Japan; 2 Center for Regional Environmental Research, National Institute for Environmental Studies16–2 Onogawa, Tsukuba, Ibaraki 305–8506Japan

**Keywords:** arsenite, soil, oxidation, PCR-DGGE, *aioA*

## Abstract

The impact of arsenite (As[III]) on the bacterial community structure and diversity in soil was determined by incubating soil slurries with 50, 500, and 5,000 μM As(III). As(III) was oxidized to arsenate (As[V]), and the microbial contribution to As(III) oxidation was 70–100%. PCR-denaturing gradient gel electrophoresis revealed that soil bacterial diversity decreased in the presence of As(III). Bacteria closely related to the family *Bacillaceae* were predominant in slurry spiked with 5,000 μM As(III). The population size of culturable As(III)-resistant bacteria was 37-fold higher in this slurry than in unspiked slurry (*p* < 0.01), indicating that high levels of As(III) stimulate the emergence of As(III)-resistant bacteria. As(III)-resistant bacteria isolated from slurry spiked with 5,000 μM As(III) were mainly affiliated with the genus *Bacillus*; however, no strains showed As(III)-oxidizing capacity. An As(III)-oxidizing bacterial community analysis based on As(III) oxidase gene (*aioA*) sequences demonstrated that diversity was the lowest in slurry spiked with 5,000 μM As(III). The deduced AioA sequences affiliated with *Alphaproteobacteria* accounted for 91–93% of all sequences in this slurry, among which those closely related to *Bosea* spp. were predominant (48–86%). These results suggest that exposure to high levels of As(III) has a significant impact on the composition and diversity of the soil bacterial community, including the As(III)-oxidizing bacterial community. Certain As(III)-oxidizing bacteria with strong As(III) resistance may be enriched under high As(III) levels, while more sensitive As(III) oxidizers are eliminated under these conditions.

Arsenic is a toxic metalloid that ranks 20^th^ in abundance in the earth’s crust and is distributed ubiquitously in various natural systems (24). Arsenic in drinking water poses the greatest threat to human health in many parts of the world, including Bangladesh and West Bengal (27, 28). Arsenic contamination has also been reported in some paddy fields located downstream of mining areas (48). The transfer of arsenic from paddy soil to rice may amplify the risk of arsenic poisoning among the inhabitants of these areas. Since arsenic contamination in ground water and soil is a serious environmental and health hazard, the effects of arsenic have received increased attention (9).

The predominant forms of arsenic in soil and water are inorganic arsenite (As[III]) and arsenate (As[V]). The toxicity, mobility, and bioavailability of arsenic depend on its oxidation state (5); therefore, it is important to examine arsenic species under various environmental conditions. Under oxic conditions, As(V) is predominant and strongly binds to various soil minerals including iron, manganese, and aluminum (hydr)oxides. In contrast, under reducing conditions, arsenic mostly occurs as As(III), which is more mobile and toxic than As(V) (3, 42). As(III) specifically adsorbs iron and amorphous aluminum (hydr)oxides (6, 13). Manganese oxide minerals are also important minerals because they readily oxidize As(III) to As(V) abiotically (31).

Microbial redox transformations of arsenic, *i.e.*, As(V) reduction and As(III) oxidation, are key reactions in the biogeochemical cycling of arsenic (30, 47). Numerous studies have revealed that microbial activity is indispensable for the reduction of As(V) to As(III) under reducing conditions (29, 45). Microbial As(III) oxidation has also been observed in various environments. Yamamura *et al.* (46) incubated five soil slurry types with 1,000 μM As(III) and found that As(III) was oxidized to As(V) microbiologically under aerobic conditions. Another study also showed the abiotic oxidation of As(III) by a poorly crystalline manganese oxide mineral occurring in soils (16). We recently reported that microbial As(III) oxidation accounted for more than 30% of total As(III) oxidation in natural paddy soils to which no exogenous arsenic was added (8). These findings suggest that the capacity for As(III) oxidation is widespread in natural soil microbial communities.

To date, a large number of As(III)-oxidizing bacteria have been isolated from diverse environments (47). Some of these bacteria are heterotrophic As(III) oxidizers and require organic compounds for growth. In contrast, certain autotrophic bacteria have the ability to derive metabolic energy for growth from As(III) oxidation (36, 39). As(III)-oxidizing bacteria are phylogenetically diverse, but all perform As(III) oxidation by means of the enzyme As(III) oxidase, which belongs to the dimethyl sulfoxide (DMSO) reductase family (10, 17). As(III) oxidase consists of a large subunit, AioA, and a small subunit, AioB. The former contains a molybdenum site and [3Fe-4S] cluster, while the latter contains a Rieske-type [2Fe-2S] site. Genes encoding As(III) oxidase have been characterized in As(III)-oxidizing bacteria from various prokaryotic groups, including *Alpha-*, *Beta-*, and *Gammaproteobacteria* (30, 34). These genes have been used to monitor As(III) oxidizers in natural environments (14, 30, 34).

It currently remains unclear whether As(III) has an impact on natural microbial communities, particularly As(III)-oxidizing bacterial communities. Quéméneur *et al.* (35) determined the diversity and structure of As(III)-oxidizing bacteria in arsenic-polluted waters collected from disused mines in France. Arsenic levels affected the structure of the *aioA*-carrying bacterial population, which consisted of members of *Alpha*-, *Beta*-, and *Gammaproteobacteria*, and the highest copy number of the *aioA* gene was found in the most polluted locations. Lami *et al.* (20) conducted soil column experiments in order to investigate the effects of the addition of As(III) (200 μM) on soil microbial communities. The 16S rRNA gene analysis revealed that diversity was the lowest in As(III)-spiked soil, while the highest diversity was observed in the initial soil. Although the diversity of *aioA* genes did not differ significantly between spiked and initial soils, the copy number of the *aioA* gene increased in As(III)-spiked soil. These findings suggest the potential impact of As(III) on the soil bacterial community and As(III)-oxidizing bacterial community. However, it remains unclear how As(III) affects the natural soil bacterial community when the same soil is exposed to different levels of As(III).

The aim of the present study was to determine the impact of As(III) on the structure and diversity of the soil bacterial community. Soil slurries were incubated with 50, 500, and 5,000 μM As(III) under oxic conditions, and As(III) oxidizing rates were determined under these conditions. PCR-denaturing gradient gel electrophoresis (DGGE) targeting the 16S rRNA genes was performed to monitor the soil bacterial community. In addition, in order to understand the structure and diversity of the As(III)-oxidizing bacterial community, *aioA* was amplified using two sets of PCR primers. The possible mechanisms responsible for bacterial community shifts induced by As(III) and the bacterial contribution to As(III) oxidation in soil were also discussed.

## Materials and Methods

### Soil

Soil was collected from the surface layer of a fallow paddy field in April 2008. It was classified as Aeric Epiaquents according to US taxonomy (43). The arsenic concentration in the soil was 39.5 mg kg^−1^ and detailed soil properties were reported by Yamaguchi *et al.* (45). The soil was passed through a 2-mm sieve under field moist conditions and stored in a refrigerator at 7°C until used.

### Incubation of soil slurries

Moist soil (0.3 g) was mixed with 30 mL of deionized water in 100-mL Erlenmeyer flasks. As(III) (NaAsO_2_, Sigma-Aldrich, Tokyo, Japan) was added from a sterile stock solution to final concentrations in the liquid phase of 50, 500, and 5,000 μM. Duplicate samples were prepared for each concentration of As(III). In some cases, the slurries were autoclaved at 121°C for 2 h in order to determine the microbial contribution to As(III) oxidation. The flasks were sealed with silicone caps and incubated at 30°C with rotary shaking at 200 rpm. The incubation was performed for 7 to 28 d until As(III) in the liquid phase disappeared completely. In order to determine whether slurry adsorbs As(V), autoclaved slurry spiked with 50 μM As(V) (KH_2_AsO_4_, Wako Pure Chemical Industries, Osaka, Japan) was similarly incubated for 7 d.

At various time intervals, soil slurry was centrifuged, and arsenic speciation in the supernatant was determined using high performance liquid chromatography (HPLC) (L-7000, Hitachi, Tokyo, Japan) with an Aminex HPX-87H ion exclusion column (Bio-Rad Laboratories, Hercules, CA, USA) under UV detection at 212 nm. In soil slurry spiked with 50 and 500 μM As(III), arsenic speciation and the total concentration of arsenic in the supernatant were determined by HPLC connected with a quadrupole inductively coupled plasma mass spectrometer (ICPMS) as described previously (8, 45) because arsenic concentrations less than 1,000 μM cannot be measured by HPLC. An Inertsil ODS-3 column (4.6 × 150 mm; GL Science, Tokyo, Japan) was used for the separation of As(III) and As(V).

### PCR-DGGE

**Fig. 1:**
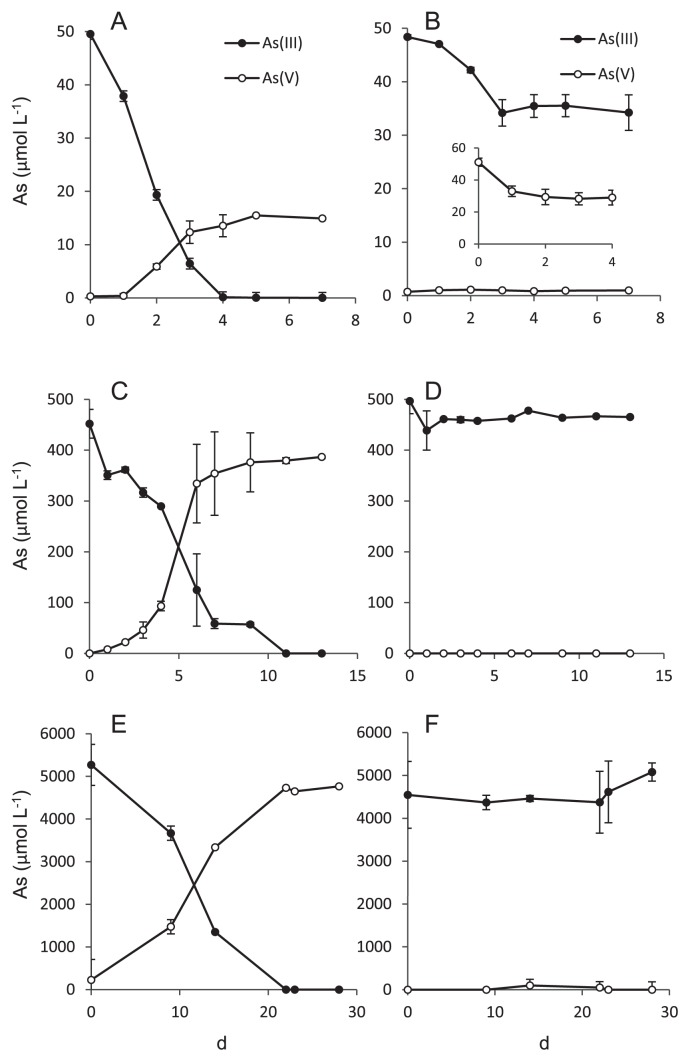
Concentrations of As(III) and As(V) in the liquid phase of soil slurries incubated with 50 (A and B), 500 (C and D), and 5,000 μM (E and F) As(III). Results from unsterilized (A, C, and E) and autoclaved (B, D, and F) slurries are shown. Symbols represent the mean values obtained for duplicate determinations, and error bars indicate the range of values. The absence of bars indicates that the range is smaller than the span of the symbol. The inset in Fig. 1B shows the adsorption of 50 μM As(V) to autoclaved slurry.

In the preliminary experiment, we extracted DNA at 11 d and 22 d in order to determine whether the incubation period had a significant impact on the soil bacterial community. As shown in Fig. S1, the community structures at these two periods were very similar, suggesting that As(III) levels rather than the incubation period have a significant impact on the soil bacterial community. Thus, we decided to extract DNA at 22 d because As(III) oxidation was still in progress at 11 d in slurry spiked with 5,000 μM As(III) (see Fig. 1E).

DNA was extracted from soil slurries using a FastDNA Spin kit (MP Biomedicals, Morgan Irvine, CA, USA) according to the manufacturer’s instructions. No duplicate extractions were performed in this study. PCR-DGGE was performed according to the method reported by Muyzer *et al.* (26), using the primers 338f+GC and 518r. The PCR protocol used was as follows: initial denaturation at 94°C for 10 min; 35 cycles of (i) denaturation at 94°C for 15 s, (ii) annealing at 55°C for 45 s, and (iii) extension at 72°C for 30 s; and final extension at 72°C for 10 min. DGGE was performed using a DCode Universal Mutation Detection System (Bio-Rad Laboratories, Hercules, CA, USA). The major bands were excised and used for reamplification with the primers 338f and 518r. PCR products were purified using a QIAquick PCR Purification kit (Qiagen, Hilden, Germany) and sequenced using a BigDye Terminator Cycle Sequencing Kit (Applied Biosystems, Foster City, CA, USA) and ABI Prism 3100 Genetic Analyzer (Applied Biosystems). The 16S rRNA gene sequences obtained were aligned to known sequences in the DNA Data Bank of Japan (DDBJ)/European Molecular Biology Laboratory (EMBL)/GenBank databases using the Basic Local Alignment Search Tool (BLAST) program (http://www.ncbi.nlm.nih.gov/BLAST/). The intensity of each band was determined using Quantity-one software (version 4.6.3, Bio-Rad Laboratories). Bacterial diversity was estimated on the basis of densitometric measurements, and the Shannon-Weaver index (*H*′) for each sample was calculated according to the equation,

$$H'=-\sum_{i=1}^{S}p_{i}1\text{n}p_{i}$$

where *S* is the total number of bands and *p_i_* is the ratio of a given band intensity to the total intensity of bands.

### Determination of culturable As(III)-resistant bacteria

Soil slurries incubated for 22 d were diluted appropriately with sterile water and spread on PTYG agar medium, which contained the following (L^−1^): peptone (2.5 g), tryptone (2.5 g), yeast extract (5.0 g), glucose (5.0 g), MgSO_4_ (0.3 g), CaCl_2_ (0.035 g), and agar (15 g). As(III) was also added at a final concentration of 5,000 μM. Medium was incubated at 30°C for 3 d, and the number of colonies that appeared was determined. Eighteen colonies were randomly selected and purified on the same medium. The 16S rRNA gene sequences of these isolates were determined as described previously (29). These isolates were inoculated into PTYG liquid medium, minimum salt medium without an organic carbon source (39), and minimum salt medium containing 0.01 to 0.1% yeast extract in order to determine whether they possess the capacity for As(III) oxidation. All of these media were supplemented with 5,000 μM As(III). During aerobic incubation at 30°C with rotary shaking at 200 rpm, arsenic speciation in the cultures was determined by HPLC as described above. In some cases, slurries were spread on PTYG agar medium containing no As(III) in order to enumerate total culturable bacteria.

### Determination of *aioA* genes

The *aioA* gene was amplified from DNA extracted from the slurries using the degenerate primers *aroA*95f (5′-TGYCABTW CTGCAIYGYIGG-3′) and *aroA*599r (5′-TCDGARTTGTASGCI GGICKRTT-3′), according to the PCR protocol described by Hamamura *et al.* (14). Other primers, *aoxB*M1-2F (5′-CCACTT CTGCATCGTGGGNTGYGGNTA-3′) and *aoxB*M3-2R (5′-TG TCGTTGCCCCAGATGADNCCYTTYTC-3′), were also used according to the PCR protocol described by Quéméneur *et al.* (34). These two primer sets amplify approximately 500 and 1,100 bp fragments of *aioA*, respectively. The PCR products were cloned into the pMD 20-T vector using the Mighty TA-Cloning Kit (Takara Bio, Otsu, Japan) and transformed into DH5α chemically competent *Escherichia coli* (Takara Bio), according to the manufacturer’s instructions. Thirty to forty clones were randomly chosen from each soil slurry and were sequenced as described previously (8). The gene sequences obtained were aligned using the Clustal W program in MEGA version 6.0 (44) and clustered into operational taxonomic units (OTU), followed by grouping at more than 97% similarity based on different distances using Mothur (http://www.mothur.org/). It is common to group arsenic-metabolizing gene (*aioA* and *arrA*) sequences at more than 97 to 98% similarity (12, 15, 25, 33). A rarefaction analysis was performed with R version 3.1.2 (http://cran.r-project.org/) and the Shannon-Weaver index was calculated as described above. In this case, *S* is the number of observed OTUs, and *p_i_* is the ratio of *aioA* sequences in a given OTU to the total number of *aioA* sequences. The gene sequences obtained were translated to amino acid sequences and subjected to a BLASTP search in order to determine sequence identity. The retrieved sequences were aligned using the Clustal X program, version 2.0. The phylogenetic tree was constructed using the neighbor-joining method (38). Bootstrap values were obtained for 1,000 replicates in order to estimate the confidence of tree topologies.

### Nucleotide sequence accession numbers

The gene sequences identified in this study have been deposited in the DDBJ/EMBL/GenBank databases under the accession numbers LC012044 to LC012307, LC027609 to LC027621, and LC051121 to LC051138.

## Results

### As(III) oxidation in soil slurries

Soil slurries were spiked with 50, 500, and 5,000 μM As(III) and incubated aerobically for 7, 13, and 28 d, respectively. In slurry incubated with 50 μM As(III), the complete disappearance of As(III) from the liquid phase occurred within 4 d (Fig. 1A). However, only 15 μM As(V) was detected in the liquid phase, suggesting that the physicochemical adsorption of As(III) or As(V) to the soil solid phase occurred together with As(III) oxidation. In autoclaved slurry, a slight decrease (15 μM) in As(III) was still observed in the liquid phase, but a significant amount of As(V) was not produced (Fig. 1B).

In slurry incubated with 500 μM As(III), As(III) disappeared completely from the liquid phase within 11 d, and the concomitant production of As(V) occurred with an As(V)/As(III) molar ratio of 0.9 (Fig. 1C). In autoclaved slurry, the significant disappearance of As(III) (approximately 30 μM) from the liquid phase was observed, without the production of As(V) (Fig. 1D). In contrast, 5,000 μM As(III) was oxidized completely within 22 d, and the equivalent production of As(V) was observed (Fig. 1E). Under this condition, the As(III) oxidation rate by soil slurry was calculated to be 22.7 μmol As(III) d^−1^ g soil^−1^. In autoclaved soil slurry, neither As(III) consumption nor As(V) production was observed (Fig. 1F), indicating that the As(III) oxidation of 5,000 μM As(III) was catalyzed microbiologically.

### PCR-DGGE analysis

**Fig. 2:**
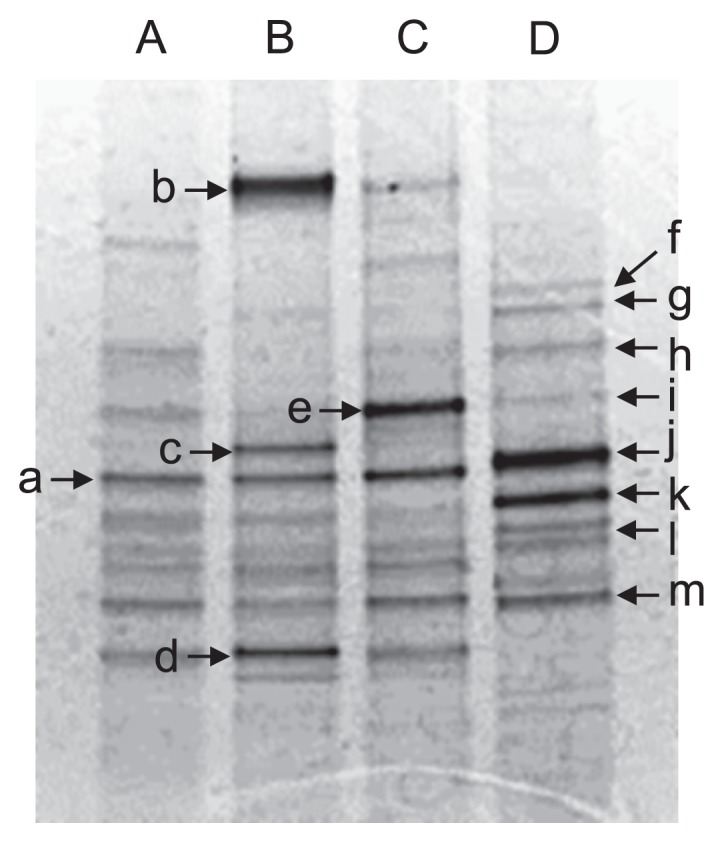
PCR-DGGE analysis of soil slurries incubated with 0 (lane A), 50 (lane B), 500 (lane C), and 5,000 μM (lane D) As(III) for 22 d. Arrows indicate bands recovered for DNA sequencing (see Table 1).

In an attempt to more fully understand the potential impact of various concentrations of As(III) on the soil bacterial community, DNA was extracted from each slurry, and a PCR-DGGE analysis was performed (Fig. 2). The major bands were excised from the DGGE gel and sequenced to determine their identities (Table 1). As shown in Fig. 2, the DGGE profiles of slurries spiked with 50 and 500 μM As(III) shared many bands with that of soil slurry incubated without As(III) (unspiked slurry); however, the bands closely related to *Chitinophaga filiformis* (band b), *Bdellovibrio bacteriovorus* (band c), *Bacillus thermocopriae* (band d), and *C. arvensicola* (band e) were newly observed. In contrast, slurry spiked with 5,000 μM As(III) exhibited a different DGGE profile (Fig. 2, lane D); bands a, b, c, d, and e, which were observed in unspiked slurry or in slurries spiked with 50 and 500 μM As(III), disappeared in this slurry. On the other hand, unique DGGE bands (bands f, g, h, i, j, k, l, and m), all of which were affiliated with the family *Bacillaceae*, were newly observed. In addition, soil bacterial diversity decreased in the presence of As(III), *i.e.*, the Shannon-Weaver indices (*H*′) of unspiked slurry and slurries spiked with 50, 500, and 5,000 μM As(III) were 2.022, 1.711, 1.511, and 1.496, respectively.

### As(III)-resistant bacteria in slurries

Diluted soil slurries were spread on PTYG agar medium in order to determine the population sizes of total culturable bacteria (Table 2). In unspiked slurry and slurries spiked with 50 or 500 μM As(III), no significant differences were observed in the population sizes of total culturable bacteria (Student’s *t*-test, *p* > 0.23, *n*=3), which were between 7.77 and 7.90 log10 CFU g soil^−1^. However, the population size was slightly lower (7.34 log10 CFU g soil^−1^, Student’s *t*-test, *p* < 0.01, *n*=3) in slurry spiked with 5,000 μM As(III).

Diluted slurries were also spread on PTYG medium containing 5,000 μM As(III) in order to determine the population sizes of culturable As(III)-resistant bacteria (Table 2). The abundance of As(III)-resistant bacteria in unspiked slurry was 6.25 log10 CFU g soil^−1^, which is only 2.3% of total culturable bacteria in this slurry. In contrast, the abundance of As(III)-resistant bacteria in slurry spiked with 5,000 μM As(III) was 7.82 log10 CFU g soil^−1^, which is similar to total culturable bacteria in this slurry, and 37-fold higher than that of unspiked slurry. In the cases of slurries spiked with 50 and 500 μM As(III), the abundance of As(III)-resistant bacteria was similar and slightly higher (Student’s *t*-test, *p* < 0.01, *n*=3) than that in unspiked slurry, respectively. In both cases, the abundance of As(III)-resistant bacteria was significantly lower (Student’s *t*-test, *p* < 0.01, *n*=3) than that of total culturable bacteria in unspiked or 50 and 500 μM-spiked slurries.

In order to determine As(III)-resistant bacteria in slurry spiked with 5,000 μM As(III), 18 colonies were randomly selected, purified, and identified based on their 16S rRNA gene sequences. As shown in Table S1, 16 out of the 18 strains were affiliated with the genus *Bacillus*. These strains were inoculated into PTYG medium, minimal medium without an organic carbon source (39), and minimal medium supplemented with 0.01 to 0.1% yeast extract to confirm whether they possess the capacity for As(III) oxidation under autotrophic or heterotrophic conditions. No strains showed significant As(III) oxidation in the presence of 5,000 μM As(III); however, all of these strains grew well under heterotrophic conditions. In order to isolate autotrophic As(III)-oxidizing bacteria directly from slurry, slurry spiked with 5,000 μM As(III) was also spread on minimal medium supplemented with 5,000 μM As(III). However, no autotrophic As(III) oxidizers were isolated.

### As(III)-oxidizing bacterial community analysis

**Fig. 3:**
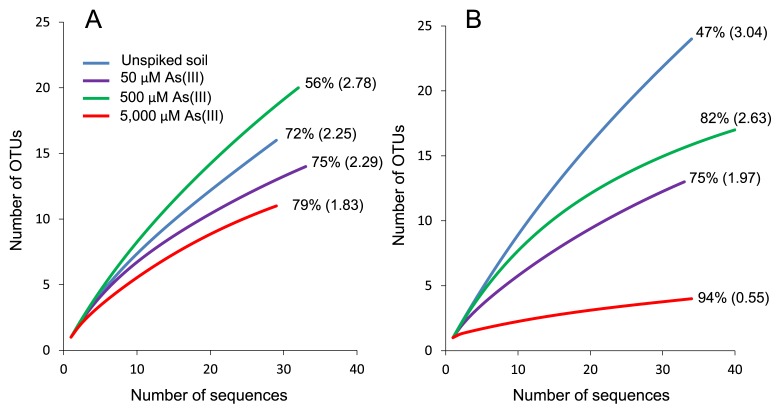
Rarefaction analysis of the *aioA* gene in soil slurries. *aioA* was amplified with either the primer set *aioA*95f/*aioA*599r (A) or *aoxB*M1-2F/*aoxB*M3-2R (B). Operational taxonomic units (OTUs) were defined at the 97% DNA sequence identity level. Percentage values represent coverage indices, which were calculated by a Chao analysis. The values in parentheses represent Shannon-Weaver indices (*H*′).

As(III)-oxidizing bacterial communities in slurries were analyzed by cloning and sequencing the *aioA* gene. We first used the primer set *aioA*95f/*aioA*599r (14), which specifically amplifies an approximately 500-bp fragment of *aioA*. *aioA* clones (29–32 clones) were sequenced and clustered into 11 to 20 OTUs (grouped at > 97% similarity) in each slurry. Rarefaction curves and coverage indices indicated that 56–75% of the total *aioA* diversity was sampled in unspiked slurry as well as in slurries spiked with 50 and 500 μM As(III) (Fig. 3A). The *H*′ values of these three slurries were 2.25, 2.29, and 2.78, respectively. In contrast, in slurry spiked with 5,000 μM As(III), *aioA* diversity was the lowest among all slurries, with a coverage index and *H*′ value of 79% and 1.83, respectively.

**Fig. 4:**
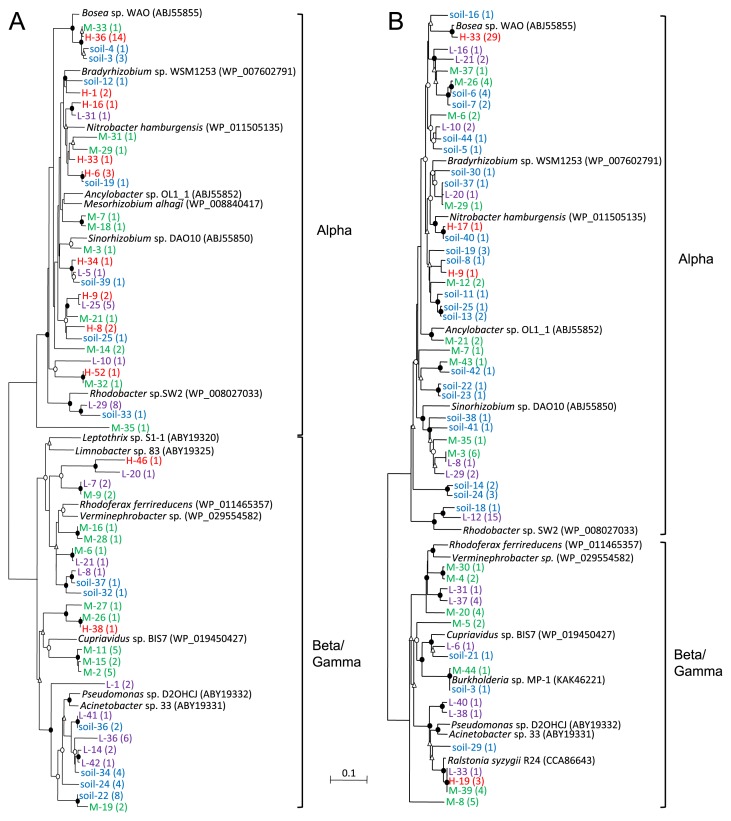
Neighbor-joining phylogenetic tree of AioA sequences found in unspiked slurry (blue) and slurries incubated with 50 (purple), 500 (green), and 5,000 μM (red) As(III). Circles and triangles at branch nodes represent bootstrap percentages (1,000 replicates): filled circles, 90–100%; open circles, 70–89%; open triangles, 50–69%. Values <50% are not shown. The scale bar represents the estimated number of substitutions per site. *aioA* was amplified with either the primer set *aioA*95f/*aioA*599r (A) or *aoxB*M1-2F/*aoxB*M3-2R (B). The values in parentheses represent the number of clones contained in each corresponding OTU (based on a 97% cut-off).

**Fig. 5:**
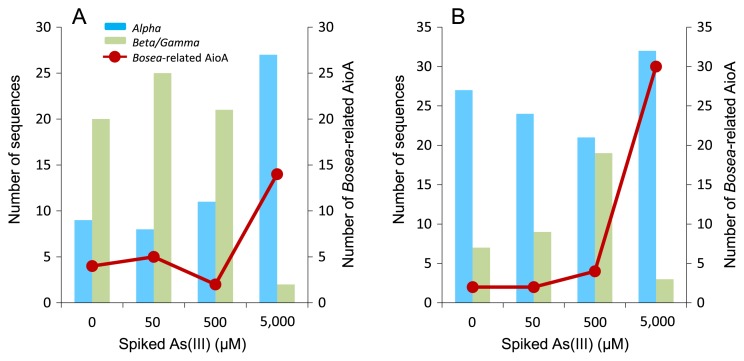
Number of AioA sequences clustered in *Alphaproteobacteria* and *Beta/Gammaproteobacteria*-related groups in unspiked slurry and slurries incubated with 50, 500, and 5,000 μM As(III). The red circles show the number of AioA sequences related to *Bosea* sp. WAO in each slurry. *aioA* was amplified with the primer set *aioA*95f/*aioA*599r (A) or *aoxB*M1-2F/*aoxB*M3-2R (B).

The *aioA* sequences obtained were translated into amino acid sequences for a taxonomic analysis (Fig. 4A). Phylogenetically diverse AioA was present in unspiked slurry as well as slurries spiked with 50 and 500 μM As(III). All of these sequences were related to those from *Alpha-*, *Beta-*, and *Gammaproteobacteria*, and many AioA sequences (20–25 clones) belonged to the *Beta/Gammaproteobacteria*-related group (Fig. 5A). In contrast, the addition of 5,000 μM As(III) had a significant impact on As(III)-oxidizing bacterial communities since the deduced AioA affiliated with *Alphaproteobacteria* became predominant (27 out of 29 clones). Among these, AioA related to *Bosea* sp. WAO accounted for 48% (14 out of 29 clones) of the total sequences in this slurry (Fig. 4A and 5A).

A similar experiment was performed with another primer set, *aoxB*M1-2F/*aoxB*M3-2R (34), which yields an approximately 1,100-bp fragment of the *aioA* gene. In each slurry, 33 to 40 sequences were obtained and clustered into 4 to 24 OTUs. Rarefaction curves and coverage indices indicated that 47–94% of total *aioA* diversity was sampled in these slurries (Fig. 3B). The *H*′ values of unspiked slurry and slurries spiked with 50, 500, and 5,000 μM As(III) were 3.04, 1.97, 2.63, and 0.55, respectively, indicating that *aioA* diversity was the lowest in slurry spiked with 5,000 μM As(III). In unspiked slurry and As(III)-spiked slurries, the deduced AioA affiliated with *Alphaproteobacteria* (21–32 clones) were predominant (Fig. 4B and 5B). AioA related to *Bosea* sp. WAO were predominant in slurry incubated with 5,000 μM As(III), which accounted for 86% (30 out of 35 clones) of the total sequences (Fig. 5B).

## Discussion

In autoclaved slurry spiked with 50 μM As(III), 15 μM As(III) disappeared from the liquid phase, indicating that As(III) was removed abiotically (Fig. 1B). Dissolved As(III) appears to have been adsorbed onto the surface of iron (hydr) oxides in the soil solid phase, which are known as superior As(III) adsorbents at neutral pH (6). Conversely, 50 μM As(III) disappeared completely from the liquid phase in slurry incubated without autoclaving (Fig. 1A). Since the abiotic adsorption of As(III) was also expected to have occurred in this slurry, 15 μM As(III) is considered to be adsorbed on the soil solid phase. The remaining 35 μM of As(III) may be oxidized to As(V) microbiologically, and, thus, the As(III) oxidation rate under this condition was calculated to be 0.875 μmol As(III) d^−1^ g soil^−1^. We also observed the abiotic adsorption of As(V) on the soil solid phase and found that autoclaved slurry removed 20 μM of As(V) from the liquid phase (Fig. 1B, inset). These results are consistent with only 15 μM As(V) being detected in the liquid phase of slurry spiked with 50 μM As(III) (Fig. 1A).

The significant adsorption of both As(III) and As(V) may have occurred in slurry spiked with 500 μM As(III), and the As(III) oxidation rate was calculated to be 3.82 μmol As(III) d^−1^ g soil^−1^. In contrast, no significant abiotic removal of As(III) was observed in slurry spiked with 5,000 μM As(III), and, thus, nearly 100% of As(III) is considered to be oxidized microbiologically.

In a previous study (8), we found that As(III) oxidation in soil slurries, without any addition of exogenous As(III), mainly occurred abiotically through the reaction with soil minerals, such as poorly crystalline manganese oxides. In this case, the maximum As(III) concentration in slurry was 3 to 4 μM, and the microbial contribution to As(III) oxidation was only approximately 30%. In the present study, the theoretical microbial contribution to As(III) oxidation was 70% with the addition of 50 μM As(III), as discussed above, and nearly 100% when 500 and 5,000 μM As(III) were added. Several other studies observed the oxidation of 200 to 1,000 μM As(III) in soils, but found no abiotic oxidation (20, 46). These findings suggest that the microbial contribution to As(III) oxidation increases as As(III) concentrations become higher. Although the abiotic oxidation of As(III) plays an important role with markedly lower concentrations of As(III) (8), its capacity is limited due to the passivation of reactive sites on the mineral surface (18, 19).

A PCR-DGGE analysis revealed that the *H*′ value in unspiked slurry (2.0) decreased gradually to 1.5 and was accompanied by an increase in As(III) concentrations, suggesting that As(III) has a negative impact on soil bacterial diversity possibly due to its toxicity. Similar findings were previously obtained by Lami *et al.* (20), who conducted soil column experiments with a natural microbial community exposed to a continuous flow of 200 μM As(III). The bacterial taxa, determined by 454 pyrosequencing of the 16S rRNA gene, decreased by nearly one-half in As(III)-spiked soil with a concomitant decrease in the *H*′ value from 7.1 in initial soil to 5.4 in As(III)-spiked soil (20).

The DGGE bands closely related to the family *Bacillaceae* were predominant in slurry spiked with 5,000 μM As(III) (Fig. 2). The number of culturable As(III)-resistant bacteria was similar to that of total culturable bacteria in this slurry (Table 2), indicating that high levels of As(III) strongly stimulate the emergence of As(III)-resistant bacteria. Most As(III)-resistant bacteria isolated from this slurry were members of the genus *Bacillus* (Table S1), which is in good agreement with the results of the PCR-DGGE analysis. However, they were not considered to play roles in As(III) oxidation in slurry because they did not show As(III)-oxidizing capacity under autotrophic or heterotrophic conditions. We also attempted to amplify *aioA* fragments from the genomic DNA of these isolates, but were unsuccessful (data not shown). Members of the genus *Bacillus* have frequently been isolated as arsenic-resistant bacteria from arsenic-contaminated ore (41), sediments (32), plant roots (4), groundwater (21), and soils (1, 23). They sometimes show minimal inhibitory concentrations for As(III) of nearly 50 mM (23). This strong resistance is likely due to the presence of arsenic detoxification proteins, including the As(III) efflux pumps ArsB and Acr3. The genes encoding these detoxification proteins have been detected in many arsenic-resistant *Bacillus* strains (1, 4, 21). It is also possible that *Bacillaceae* bacteria became predominant in As(III)-spiked slurry due to the ability of this group to form physiologically resistant structures, *i.e.*, endospores (23).

*aioA* gene diversity in slurries spiked with 50 and 500 μM As(III) (*H*′ values of 1.97 to 2.63) was lower than that in unspiked soil (*H*′ of 3.04) when the primer set *aoxB*M1-2F/*aoxB*M3-2R was used. However, conflicting results were obtained when another primer set (*aioA*95f/*aioA*599r) was used; the *H*′ values of As(III)-spiked slurries (2.29 to 2.78) were similar to those of unspiked slurry (2.25). The rarefaction curves and coverage indices of these slurries showed under-saturation (Fig. 3), indicating that a stronger sequencing effort is needed in order to completely define diversity in these slurries. In contrast, in slurry spiked with 5,000 μM As(III), *aioA* gene diversity determined with the primer sets *aoxB*M1-2F/*aoxB*M3-2R and *aioA*95f/*aioA*599r was the lowest (*H*′ values of 0.55 and 1.83, respectively) with relatively high coverage indices of 94% and 79%, respectively. The deduced AioA sequences affiliated with *Alphaproteobacteria* accounted for 91–93% of the total sequences in this slurry, among which those closely related to *Bosea* sp. WAO were predominant (48–86%, Fig. 4 and 5). Alphaproteobacterial As(III)-oxidizing bacteria, including *Bosea* sp. WAO, have frequently been isolated from various environments (7, 11, 22, 37, 39, 40). Many of these bacteria are facultative chemolithoautotrophic As(III) oxidizers and are highly resistant to As(III) levels of more than 5,000 μM. In addition, they are capable of growing rapidly, even in the presence of more than 5,000 μM As(III). For example, strain NT-26 isolated from a gold mine grew autotrophically in the presence of 5,000 μM As(III) with a doubling time of 7.6 h (39). Therefore, it is possible that highly resistant As(III)-oxidizing bacteria such as *Bosea* spp. increased or maintained their population sizes in slurry spiked with 5,000 μM As(III), whereas more sensitive or slow-growing As(III) oxidizers were eliminated under this condition. As(III) resistance and As(III) oxidation rates are known to vary significantly between As(III)-oxidizing bacteria (2). Our results suggest that exposure to high As(III) levels has a significant impact on the structure and diversity of the As(III)-oxidizing bacterial community in soil, and that certain As(III)-oxidizing bacteria with strong As(III) resistance are enriched specifically under these conditions. The complete oxidation of 5,000 μM As(III) generally yields sufficient metabolic energy for growth under chemolithoautotrophic conditions (7, 22, 37, 39). Hence, we speculate that *Bosea-*like As(III)-oxidizing bacteria also increased their biomass in slurry spiked with 5,000 μM As(III). Alternatively, *Bosea-*like As(III)-oxidizing bacteria may oxidize As(III) under heterotrophic conditions by using organic compounds included in soil slurry (7, 22, 37, 39). In this case, As(III) oxidation may be a detoxification process, but not an energetic process. A quantitative determination of the abundance of the *aioA* gene in slurries is required in order to further understand the roles and behavior of As(III)-oxidizing bacteria with high levels of added As(III).

## Conclusion

We herein demonstrated that As(III) had a significant impact on the composition and diversity of the soil bacterial community, including the As(III)-oxidizing bacterial community. Since As(III)-oxidizing bacteria play a pivotal role in arsenic transformation in soils, and, thus, have a significant influence on the fate and behavior of arsenic in terrestrial environments, it is of great importance to understand the possible impact of As(III) on the natural As(III)-oxidizing bacterial community. Our results suggest that certain As(III)-oxidizing bacteria are enriched even when extremely high levels of As(III) are present. Since they effectively oxidize As(III) to less toxic and relatively immobile As(V), microbial As(III) oxidation may play a key role in arsenic mobility in various environments.

## Supplementary Material



## Figures and Tables

**Table 1:** Sequence analysis of 16S rRNA genes recovered from DGGE bands.

Band	Length (bases)	Most closely related organisms in the GenBank database	Accession no.	Similarity (%)
a	143	*Bacillus acidiceler*	KF475796	98
b	154	*Chitinophaga filiformis*	NR_113724	91
c	113	*Bdellovibrio bacteriovorus*	AF148941	94
d	135	*Bacillus thermocopriae*	KP010245	99
e	153	*Chitinophaga arvensicola*	AB681052	92
f	137	*Psychrobacillus psychrodurans*	KF958479	94
g	132	*Psychrobacillus psychrodurans*	KF958479	94
h	124	*Psychrobacillus psychrodurans*	KF535154	98
i	122	*Bacillus licheniformis*	KM287418	96
j	162	*Bacillus licheniformis*	KM287420	95
k	142	*Psychrobacillus psychrodurans*	KF958479	97
l	128	*Psychrobacillus psychrodurans*	KF958479	95
m	135	*Bacillus niacini*	AY167817	96

**Table 2:** Population sizes of total culturable bacteria and As(III)-resistant bacteria in soil slurries.

Spiked As(III) (μM)	Log10 CFU g soil^−1^ on PTYG medium containing:	

	0 μM As(III)^a^	5,000 μM As(III)^b^
0 (unspiked slurry)	7.88 ± 0.71^c^	6.25 ± 0.24
50	7.90 ± 0.07	6.58 ± 0.27
500	7.77 ± 0.11	6.74 ± 0.08
5,000	7.34 ± 0.06	7.82 ± 0.02

a Slurries were spread on PTYG medium containing no As(III) in order to determine total culturable bacteria.

b Slurries were spread on PTYG medium containing 5,000 μM As(III) in order to determine culturable As(III)-resistant bacteria.

c Values represent the mean of triplicate experiments.
