# Supplementary material for: Impact of Arsenite on the Bacterial Community Structure and Diversity in Soil
Source: Microbes Environ. 2016 Feb 20;31(1):41–8. doi: 10.1264/jsme2.ME15093 (PMC4791115; doi:10.1264/jsme2.ME15093)
Supplement: Supplementary file 1 [file 31_41_s1.pdf]

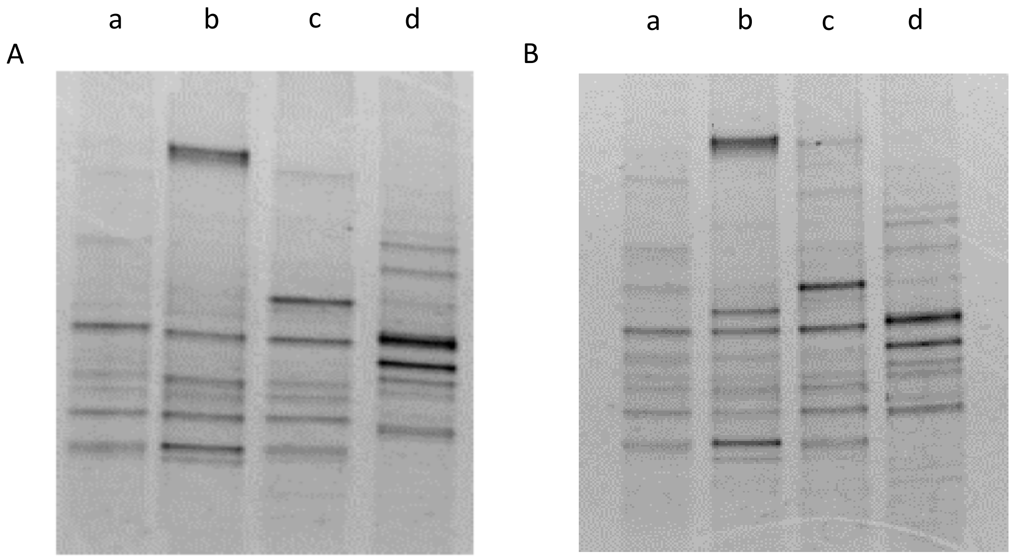

Fig. S1. PCR-DGGE analysis of the soil slurries spiked with 0 (lane a), 50 (lane b), 500 (lane c), and 5,000 μM As(III) (lane d) incubated for 11 days (A) and 22 days (B).

Table S1. 16S rRNA genes of As(III)-resistant bacteria isolated from the slurry incubated with 5,000 μM As(III)

Isolates	Length (bases)	Most closely related organisms in GenBank database	Accession no.	Similarity (%)
1	136	*Bacillus thioparans*	KM374719	100
2	140	*Bacillus niacini*	AY167817	99
3	134	*Bacillus niacini*	AY167817	99
4	138	*Bacillus thioparans*	KM374719	93
5	134	*Bacillus niacini*	AY167817	100
6	134	*Bacillus niacini*	AY167817	99
7	134	*Bacillus licheniformis*	KM505054	99
8	128	*Rhodococcus erythropolis*	GU944896	98
9	140	*Bacillus niacini*	AY167817	99
10	141	*Bacillus niacini*	AY167817	98
11	138	*Bacillus niacini*	AY167817	99
12	138	*Bacillus niacini*	AY167817	99
13	148	*Bacillus niacini*	AY167817	97
14	126	*Rhodococcus equi*	AB999844	98
15	135	*Bacillus niacini*	AY167817	99
16	132	*Bacillus niacini*	AY167817	98
17	133	*Bacillus niacini*	AY167817	99
18	138	*Bacillus niacini*	AY167817	99
